# Cost of Chronic and Episodic Migraine: a pilot study from a tertiary headache centre in northern Italy

**DOI:** 10.1186/s10194-015-0532-6

**Published:** 2015-05-27

**Authors:** E Berra, G Sances, R De Icco, M Avenali, M Berlangieri, I De Paoli, M Bolla, M Allena, N Ghiotto, E Guaschino, S Cristina, C Tassorelli, G Sandrini, G Nappi

**Affiliations:** Headache Science Centre, “C. Mondino” National Neurological Institute, Pavia, Italy; Department of Brain and Behavior, University of Pavia, Pavia, Italy; Neurological and Orthopedic Rehabilitation Unit, Clinica “Villa Esperia”, SaliceTerme, Italy

**Keywords:** Migraine, Chronic migraine, Episodic migraine, Cost, Resource utilization, Italy

## Abstract

**Background:**

Chronic migraine (CM) has a high impact on functional performance and quality of life (QoL). CM also has a relevant burden on the National Health Service (NHS), however precise figures are lacking. In this pilot study we compared the impact in terms of costs of CM and episodic migraine (EM) on the individual and on the National Health System (NHS). Furthermore, we comparatively evaluated the impact of CM and EM on functional capability and on QoL of sufferers.

**Methods:**

We enrolled 92 consecutive patients attending the Pavia headache centre: 51 subjects with CM and 41 with EM. Patients were tested with disability scales (MIDAS, HIT-6, SF-36) and with an *ad hoc* semi-structured questionnaire.

**Results:**

The direct mean annual cost (in euro) per patient suffering from CM was €2250.0 ± 1796.1, against €523.6 ± 825.8 per patient with EM. The cost loaded on NHS was €2110.4 ± 1756.9 for CM, €468.3 ± 801.8 for EM. The total economic load and the different sub-items were significantly different between groups (CM vs. EM p = 0.001 for each value).

CM subjects had higher scores than EM for MIDAS (98.4 ± 72,3 vs 15.5 ± 17.7, p = 0.001) and for HIT-6 (66.1 ± 8.4 vs 58.7 ± 10.1, p = 0.001). The SF-36 score was 39.9 ± 14,74 for CM and 66.2 ± 18.2 for EM (p = 0.001).

**Conclusions:**

CM is a disabling condition with a huge impact on the QoL of sufferers and a significant economic impact on the NHS. The adequate management of CM, reverting it back to EM, will provide a dual benefit: on the individual and on the society.

## Background

Prevalent pain conditions impose a significant economic impact on individuals and on society. Migraine represents a common pain disorder, with a prevalence in the general population of 11 % [1]. The incidence of migraine is higher in women with a peak during productive years (between 25 and 55 years of age) in Western countries [1, 2], which translates into very high costs of migraine, for both the individual and the society. As a consequence, migraine is ranked among the most costly neurological diseases for European society [2, 3]. Available literature mostly refers to the episodic form of migraine, while less is known about the economic impact of chronic migraine (i.e. headache present in more than 15 days/month) [4], whose prevalence varies from 0.5 to 5.1 % of the general population [5, 6].

“Cost of illness” represents an economic evaluation methodology and it allows to quantify the cost caused by illnesses on the population [7]. This economic evaluation usually considers two categories of costs: direct and indirect costs. Direct costs are attributable to the delivery of medical care and include medications costs, medical exams and physician and hospital visits. Compelling evidence indicates that patients with headache in primary care cost at least 87 % more than their similar-age and same gender counterparts without headache [8]. Indirect costs include impact on functional capacity, resulting in reduction of work and social activities induced by illness. Indirect costs are often undervalued but they indeed represent a problem of vast proportions, both in terms of quality of life for individuals, and in terms of economic loss for society.

Published data suggest that the economic impact is higher for patients with the chronic pattern of headache (CH) than for patients with episodic forms in United States and in Europe. However, the estimated cost related to headache presents a great variability in the different studies [9–11]. This variability is due to the different methods performed and partly to the countries and to the time period in which studies were conducted.

The main aim of this pilot study was the comparison of the impact in terms of costs of CM and episodic migraine (EM) on the individual and on the National Health System (NHS). Furthermore, we comparatively evaluated the impact of CM and EM on functional capability and on QoL of sufferers.

## Methods

The study is a cross-sectional cost-of-illness survey, conducted on patients with Chronic and Episodic Migraine attending the Headache Centre of the National Neurological Institute Casimiro Mondino Foundation of Pavia, Italy. Data was collected by means of an *ad hoc* questionnaire developed by the Authors and formed by18 items subdivided into 4 sections: demographic and general health and headache-related data, impact of headache on school, work and leisure activities, use of medication (acute or preventive treatments) and use of health resources (consultations, investigations, and hospitalizations). The questions were mostly categorical, although a few of them were open-text.

### Diagnostic methodology

Patients were first evaluated by two expert neurologists (GS, NG), who collected clinical parameters and defined the diagnosis. Subsequently, patients were asked to fill in the questionnaire with the help of a young doctor (RDI). The same doctor also administered the following scales aimed at investigating the impact of headache on function and quality of life: MIDAS (Migraine Disability Assessment) assesses headache-related disability in the past three month and is the most frequently used disability instrument in migraine research and clinical practice [12]; HIT-6 (The Headache Impact Test) examines impact of headache on limitation of daily performances [13], and SF36-tm (Health Survey) investigates the quality of life [14].

### Study subjects

The questionnaire was administered to consecutive subjects with CM (case group) and EM (control group) who attended the Pavia Headache Centre between September and December 2013 and who met the inclusion criteria listed below. The diagnoses were based on the criteria of the International Classification of Headache Disorders, III Edition (ICHD-IIIbeta) [4].

### Inclusion criteria

1. Male and female patients aged between 18 and 65 years;
2. Diagnosis of Episodic migraine or Chronic Migraine according to the ICHD-IIIbeta;
3. Age at migraine onset < 50 years;
4. Absence of any other neurological disorders;
5. Capability to differentiate headache attacks that are not migraine;
6. Capability and willingness to fill out the questionnaire and to give their informed consent

### Patients were excluded from the study in the case of one or more of these conditions

1. Ongoing pregnancy or nursing;
2. Severe cardiovascular, respiratory, metabolic or psychiatric disorders;
3. Neurological disorders different from migraine;
4. Any other disease or conditions that could compromise the patient’s participation in the study;
5. Abuse of alcohol or other drugs;
6. Use of NSAIDs or antiepileptic drugs or antidepressant drugs, a daily basis, for other disorders different from migraine.

The local Ethics Committee approved the general protocol of survey and the data management.

All participants received and signed an informed consent to participate in the study.

### Data analysis

Disability associated to migraine was calculated by means of MIDAS and of HIT-6. More specifically, MIDAS was used to calculate number of days lost due to illness [12], while HIT-6 allowed to evaluate the global impact of migraine in daily performances [13]. Subjective perception of quality of life was investigated by means of the SF-36 scale [14].

Direct costs were collected and aggregated in five categories: symptomatic medications, prophylactic medications, clinical consultations, diagnostic investigations and hospitalizations.

Symptomatic and prophylactic medications can be either partially subsidized by the National Health System (NHS) or not subsidized at all, depending on pharmaceutical classes. Their cost was calculated by considering each separate class and quantifying the amount of the subsidization and the contribution required to the patient. Consultations and diagnostic investigations are partially subsidized by NHS, their cost was therefore calculated by considering separately the amount of the subsidization provided by NHS and the contribution required to the patient, as derived from the national and regional formularies [15]. Hospitalizations are entirely subsidized by the NHS and reimbursed according to Diagnosis Related Group (DRG), a system, introduced in Italy from 1995, that allows to classify all patients discharged from a hospital in homogeneous groups for absorption of resources committed. This aspect makes it possible to quantify this cost absorption of resources and thus remunerate each episode of hospitalization, in order to control health care costs [16].

Acute and prophylactic medications used during the previous 3 months were adjusted in order to reach an estimate of the gross annual cost. Cost of medications was identified from a private site for health care professionals [17].

### Statistical analysis

The minimum sample size of the study was estimated with the “Open Source Epidemiologic Statistics for Public Health” [18]. Basing on our expertise and after an extensive literature search, we considered meaningful a difference in total annual cost between CM and EM of at least €300. So the sample size was calculated using the following parameters: confidence interval (two-sided) 95 %; power 95 %; ratio of sample size 1; mean difference €300; standard deviation €400 for CM and €350 for EM (to take in account intra-group high variability).

The suggested minimum sample size was 82 (41 patients per group).

The Statistical Package for the Social Sciences (SPSS) for Windows, version 21.0, was used for the statistical analysis.

The normality of distribution of all our variables was assessed in terms of “skewness”, “excess kurtosis” and z-tests for both skewness and kurtosis. In addition, the data were plotted using “Q-Q plot” to visually confirm normality. All data showed a Gaussian distribution, so parametric tests were chosen for the analysis.

Categorical variables were plotted in cross-tables, and statistical significance was performed with chi-square test. Regarding continuous variables, differences between the two study groups (CM vs. EM) were tested with Student’s *t*-test for unpaired groups. To enlighten intra-group statistically significance between Total costs, NHS costs and private costs we used an ANOVA (analysis of variance) test, with post hoc Bonferroni’s correction.

The level of significance was set at 0.05 (always corrected if necessary) for all variables.

## Results

We enrolled 92 patients, 51 subjects with CM and 41 subjects with EM, who consecutively attended the Headache Centre of the National Neurological Institute Casimiro Mondino Foundation of Pavia, Italy. Of the 51 CM subjects, 48 were overusing symptomatic medications and they also fulfilled criteria for medication overuse headache (MOH).

Demographic and clinical features of participants are summarized in Table 1.

**Table 1:** Demographic and clinical features of partecipants with Chronic (CM) and Episodic Migraine (EM)

	CM	EM	p-value
Age	46.9 ± 9.6	48.2 ± 13.8	Ns
Sex (F/M)	45/6	30/11	Ns
Age at migraine onset	21.5 ± 8.7	23.1 ± 11.9	Ns
Duration of chronic migraine (years)	4.42 ± 7.9	-	-
Days of migraine/month	21.3 ± 3.3	4.4 ± 3.1	0.001
Days of drug intake/month	20.2 ± 5.7	4.1 ± 2.6	0.001
Number of drugs intake/month	27.1 ± 20.6	4.2 ± 4.1	0.001

### Annual direct cost per person

When considering the 92 patients as a whole, the mean direct cost per year was €1480.7 ± 1678.2 euros, most of which (€1378.6 ± 1628.5, 93.1 %) was covered by NHS subsidization.

Hospitalizations accounted for 93 % of this cost (€797.1 ± 1166.1). The mean cost for clinical consultation was lighter €101.4 ± 136.5, and it was covered for the 59.3 % (€60.16 ± 62.2) by NHS.

The cost for investigations was €108.1 ± 172.8, covered by NHS for the 79.7 % (€86.2 ± 137.7).

The mean cost of treatments was €474.1 ± 886.6, which was mostly covered by NHS (€413.3 ± 870.8, 87.1 %).

The cost of treatment was split in: symptomatic medications for most of it with €370.8 ± 781.8, of which €335.3 ± 769.7 (90.4 %) covered by the NHS, and prophylactic therapies with €103.3 ± 347.0, of which €77.9 ± 344.8 (75.4 %) covered by NHS.

The separate analysis of costs associated with the episodic and chronic forms, showed a total annual direct cost was of €2250.1 ± 1796.1 euros per subject with CM and €523.6 ± 825.8 per subjects with EM. The burden sustained by NHS corresponded to 93.7 % of the total amount, i.e. €2110.4 ± 1756.9 euros for CM patients, and to 89.4 % of the total sum (i.e. €468.3 ± 801.8 euros) for EM subjects.

The mean per patient cost for hospitalizations was of €1242.9 ± 1251.2 for CM and €242.6 ± 753.5 euros for EM. The mean per patient cost for clinical consultations was €131.8 ± 163.7 for CM and €63.5 ± 79.1 for EM. NHS covered €69.2 ± 67.9 (52.5 %) and €48.8 ± 52.9 (76.8 %), respectively.

The cost for diagnostic investigations was of €113.3 ± 163.6 for CM group covered for 85.6 % by NHS (96.9 ± 140.1), 101.6 ± 185.4 for EM group, covered for 72.3 % by NHS (€73.4 ± 133.5).

The mean cost of treatments was €762.1 ± 1105.1 for CM group and €115.9 ± 15.2 for EM group. NHS contributed €685.1 ± 1094.1 (89.9 %) in the CM group and €75.2 ± 133.8 (65.2 %) in the EM group.

The cost of treatment was mostly represented by symptomatic medications in the CM group, where it amounted to €624.3 ± 980.0 (of which 90.8 %, i.e. 567.7 ± 974.5 covered by the NHS). In the EM group, the cost for symptomatic medications was €55.3 ± 78.2 (of which 83.5 %, i.e. €46.2 ± 79.5 subsidized by the NHS).

All the data, with intra- and inter-groups statistical significances, are summarized in the Table 2.

**Table 2:** Annual direct cost per patient

		TOTAL AVERAGE BURDEN	CM	EM	p-value (CM vs. EM)
TOTAL COSTS	TOTAL	1480.7 ± 1678.2	2250.1 ± 1796.1	523,6 ± 825.8	0.001
	NHS	1378.6 ± 1628.5	2110.4 ± 1756.9	468,3 ± 801.8	0.001
	PRI	102.1 ± 151.1	139.6 ± 181.1	55,3 ± 83.2	0.007
HOSPITALIZATIONS	NHS	797.1 ± 1166.1	1242.9 ± 1251.2	242.6 ± 753.5	0.001
CLINICAL CONSULTATIONS	TOTAL	101.4 ± 136.5	131.8 ± 163.7	63.5 ± 79.1	0.016
	NHS	60.16 ± 62.2	69.2 ± 67.9	48.8 ± 52.9	Ns
	PRI	41.2 ± 110.7	62.5 ± 139.5	14,6 ± 47.7	0.038
DIAGNOSTIC INVESTIGATIONS	TOTAL	108.1 ± 172.8	113.3 ± 163.6	101.6 ± 185.4	Ns
	NHS	86.2 ± 137.7	96.9 ±140.1	73.4±133.5	Ns
	PRI	21.9 ± 39.6	16.4 ± 18.7	28.2 ± 62.9	Ns
SYMPTOMATIC MEDICATIONS	TOTAL	370.8 ± 781.8	624.3 ± 980.0	55,3 ± 78.2	0.001
	NHS	335.3 ± 769.7	567.7± 974.5	46,2 ± 79.5	0.003
	PRI	35.4 ± 77.0	56.6 ± 93.4	9,0 ± 10.8	0.001
PROPHYLACTIC DRUGS	TOTAL	103.3 ± 347.0	137.6 ± 452.3	60,6 ± 121.9	Ns
	NHS	77.9 ± 344.8	117.3 ± 451.4	28,9 ± 106.1	Ns
	PRI	25.5 ± 64.3	20.3 ± 60.1	31,6 ± 69.6	Ns

CM = Chronic Migraine, EM = Episodic Migraine, NHS = National Health System, PRI = private

### Disability

When considering the 92 patients as a whole, the mean days of missed work/school were 35.1 ± 71.2 per year, whereas the mean days in which the patients’ productivity at work/school was reduced by half or more, because of headache, were 51.6 ± 82.8 per year.

CM group reported 57.2 ± 87.6 days per year of missed work/school and 82 ± 97.6 days per year with productivity at work/school reduced by half or more because of headache. The subjects with EM, in the last year, missed 6.8 ± 18 days of work/school (CM vs. EM p = 0.001), and their productivity was reduced by half or more in 13.6 ± 31.2 days of work/school (CM vs. EM, p = 0.001).

Considering all patients, the mean score for MIDAS was 61.5 ± 68.8, the mean HIT-6 score was 62.8 ± 9.8 and the mean score for the “General Health SF-36” questionnaire was 51.8 ± 20.9.

The mean score for MIDAS was 98.4 ± 72.3 for CM and 15.5 ± 17.7 for EM (CM vs. EM p = 0.001). The mean HIT-6 score was 66.1 ± 8.4 for CM and 58.7 ± 10.1 for EM (CM vs. EM p = 0.001). The mean score for the “General Health SF-36” questionnaire was 39.9 ± 14.7 for CM and 66.2 ± 18.2 for EM (CM vs. EM p = 0.001).

All the data, with intra- and inter-groups statistical significances, are summarized in the Table 3.

**Table 3:** Indirect costs and scale scores

	GLOBAL POPULATION	CM	EM	p-value (CM vs. EM)
Days of missed work/school per year	35.1 ± 71.2	57.2 ± 87.6	6.8 ± 18.0	0.001
Days with productivity reduced by half	51.6 ± 82.8	82 ± 97.6	13.6 ± 31.2	0.001
MIDAS	61.5 ± 68.8	98.4 ± 72.3	15.5 ± 17.7	0.001
General Health – SF 36	51.8 ± 20.9	39.9 ± 14.7	66.2 ± 18.2	0.001
HIT-6	62.8 ± 9.8	66.1 ± 8.4	58.7 ± 10.1	0.001

CM = Chronic Migraine, EM = Episodic Migraine

## Discussion

The present study defines and characterizes the economic burden of migraine in a patient population attending an Italian III level headache center by providing specific quantification of the cost associated to the episodic and chronic forms of disease.

Numerous studies conducted on American and European populations have shown a higher economic impact of chronic headache than episodic headache. Few of them have focused the attention on CM. The American Migraine Prevalence and Prevention (AMPP) Study [19] was a 5-year, national, longitudinal survey that involved a large population of migraineurs (14,544 subjects) with the aim to quantify the cost of transformed migraine (TM), described as a chronic headache (with and without medication overuse), evolving from EM. A total of 359 patients with EM developed TM over the 5-year observation period. The survey showed that the average per-person annual total costs, including direct and indirect costs, were 4.4-fold greater for TM ($7750) compared with those who remained episodic ($1757); per-person annual indirect costs, calculated as lost productive time, accounted for the majority of cost in TM ($ 5392; in EM: $977) [19]. In this survey the data were obtained by means of a self-administered questionnaire.

The International Burden of Migraine study (IBMS) was a cross-sectional, web-based survey on a worldwide population that collected data from 9.160 individuals with EM and 555 with CM in US and Canadian population [20] and from 5398 individuals with EM and 277 individuals with CM in European countries (UK, France, Germany, Italy, and Spain) [21]. Participants were selected assessing the International Classification of Headache Disorders, 2nd Edition (ICHD-2) diagnostic criteria for migraine and were classified into chronic or episodic migraine subgroups using headache frequency data. The ICHD-II criteria for CM were modified based on available data. Indirect costs were not calculated directly, but were estimated based on the replies contained on a questionnaire for disability and quality of life. In the US population, the total mean headache-related direct costs for participants with CM was $1035 over 3 months compared to $383 for patients with EM; the cost resulted lower in the Canadian population ($471 for CM; $172 for EM). In American and Canadian population, the IBMS study showed that there could be a difference in costs between Countries related to different medical approaches. For example, there was a superior preventive medication use in the American population as compared to the Canadian population, especially for some types of preventive drugs, i.e. antidepressant and cardiovascular drugs; in American population, more patients with episodic migraine were treated with a preventive therapy, and more patients with chronic migraine were submitted to diagnostic testing than Canadian patients [20].

Also in European countries CM participants had higher level of disability and had more provider neurological consultations, emergency department/hospital consultations and diagnostic investigations; the total mean medical costs were about three times higher for CM than EM. Total mean headache-related direct costs over 3 months were in UK €929 in CM compared to €216 in EM, in France €394 in CM compared to €121 in EM, in Germany €373 in CM compared to €174 in EM, in Italy €662 in CM compared to €207 in EM, in Spain €667 in CM compared to €273 in EM. So, among participants with CM, the average healthcare costs over 3 months varied greatly, ranging from €373 in Germany to €929.6 in the UK. The difference in mean total healthcare costs between CM and EM per 3 months in Germany was only €199.8 compared to €713.0 in the UK, €273.0 in France, €454.9 in Italy and €394.4 in Spain. The mean cost of care varied widely between countries, and these results suggest that there are differences across the five European countries included in this analysis due to a different migraine management and perhaps to different organization of NHS and reimbursements. The percentage of CM participants reporting one or more hospitalizations for migraine was more than twice for the UK (8.80 %) compared to any other country (0 % for France, 3.8 % for Germany, 3.6 % for Italy, and 3.6 % for Spain). EM participants had higher rates of symptomatic medication use in UK and Germany and higher rates of preventive medication use in Italy and Germany. The proportion of CM participants reporting occipital nerve block procedures was notably higher in the UK compared to other countries. Less than one third of CM participants in any country reported use of preventive medications, highlighting that many participants with CM are not receiving therapy, which may be beneficial [18].

Country differences in direct and indirect costs of headache have also been demonstrated in other European surveys. The Eurolight Project [11] is a cross sectional survey conducted in eight European countries; in this study, 8412 self-administered questionnaires were collected and data related to per-person annual direct and indirect costs were obtained. Estimates of indirect costs used average gender-specific salary levels in industry and services in each country, obtained from the Eurostat database [22], and one day wage was counted as the average gross annual earnings divided by 220 working days.

However the Project does not provide specific data on CM, but only on chronic headache (CH) with/without medication overuse. As regards EM, the mean per-person annual cost was €1222, with an important variability between European countries: in Italy €1034, Austria €885, Lithuania €297, Luxembourg €1446, Netherlands €1524 and Spain €1425.

The present study is conducted in an Italian III-level Headache Centre and involves a relatively small number of subjects. At variance from other studies on large populations, where the diagnosis and the data collection were derived from a questionnaire, here the diagnosis is based on the accurate clinical evaluation by an expert neurologist and clinical and economical data were obtained with a mixed methodology that involves the use of an hoc questionnaire and the direct interview/support of a young physician. Furthermore, in this paper we provide a picture of the burden of disease in a group of subjects that are affected by the condition enough to seek care. Most of the questionnaire-based surveys conducted in large populations are simply based on ICHD criteria – which only requires 5 attacks of migraine for the diagnosis- and therefore include also subjects suffering from migraine with a low recurrence.

In this frame of well-characterized migraineurs, our results confirm that chronic migraine exacts an higher economic toll compared with episodic migraine: the total mean annual per-person direct cost is € 2250 ± 1796 for CM and € 523 ± 825 for EM.

Our study was conducted only in Italy, so it was possible to calculate, in the framework of the direct costs, both cost paid by the National Health System (NHS) and cost charged to the patient.

In most American and European studies this analysis was not conducted; regulations regarding drugs prescription and subsidization by the National Health System vary greatly between Countries and this condition prevents to estimate subsidized/private cost in international multicenter studies.

Our results indicate that CM exacts a higher economic toll in direct costs both on patients and, even more so, on the Italian NHS. In the case of CM this accounts for € 2110,4 ± 1756,9 per patient/year, against € 139,6 ± 181,1 paid by patients themselves. The difference in annual costs of symptomatic treatments between CM and EM resulted statistically significant when analysing either NHS or patients’ share, with a total load of €624,3 ± 980,0 for CM (€567,7 ± 974,5 on NHS, €56,6 ± 93,4 on patients) and of € 55,3 ± 78,2 for EM (€46,2 ± 79,5 on NHS, €9,0 ± 10,8 on patients).

Also the cost of prophylactic therapy was higher in CM than EM, but not statistically significant (€137,6 ± 452,3 for CH and € 60,6 ± 121,9 for EH). This finding seems to suggest that prophylactic medications might be underused in CM subjects.

Costs for outpatients care are higher in CM, probably because patients with a high frequency of attacks require to effect more frequently visits. The similar cost observed between patients’ groups in terms of investigation is only apparently surprising, since most CM patients attending our Centre reported a chronification of migraine from several years. This means that most expensive investigations performed to rule out biological cause have been performed before the observation period considered in our study (last year).

About indirect costs, our results confirm published evidences that suggest the considerable impact of chronic migraine on functional capacity. Recent studies have demonstrated that, in Occidental countries, the loss of productivity caused by headache is one of the most important problems of chronic headache and could represents more than 50 % of the total annual costs related to headache. In some of these studies, conducted on large patient populations, national and international impact of indirect costs on the total annual economic burden was calculate. Eurolight Project showed that the indirect costs constituted 92 % of total costs in both tension-type headache and migraine. In patients with tension-type headache, indirect costs were primarily attributable to reduced productivity at work; in patients with migraine, they were attributable equally to the reduced productivity and to the number of days of absence to work [11]. In American population costs attributable to lost productive time accounted for the majority of the total cost for patients with migraine (55.7 %) and to an even greater extent for subjects who developed transformed, chronic migraine (69.6 %).

As regards the impact of disease in terms of QoL, in our study CM patients showed worse levels of physical functioning, role limitation, fatigue and loss of energy, social functioning and emotional status. Disrupted work and social activities were calculates as number of days of missed work/school and days in which patient’s productivity at work/school was reduced by half; both results showed a significantly higher impact of CM (57.2 ± 87.6 days per year of missed work/school and 82 ± 97.6 days per year of reduced productivity) when compared with EM (6.8 ± 18 days of missed work/school and 13.6 ± 31.2 days of reduced productivity; CM vs EM, p < 001).

### Limitations of the study

The present study was conducted in a single Italian III-level Headache Centre and on a relatively small number of subjects. Considering the differences, in terms of rates of treatment programs and organizational management between different Italian regions, the study provides information that cannot be generalized to the entire Country. For a national quantification of costs of CM and EM, a multicentric study conducted in different regional headache centers is necessary.

As regards direct costs, our approach was not devised for capturing costs of disorders that may be secondary to headache or to its treatment (as gastrointestinal, hepatic and renal damage caused by analgesic overuse) or comorbid; including these conditions, costs would of course increase even further.

Furthermore, a limitation of this study is that indirect economic costs were not analytically calculated. A gross estimate could have been calculated using the specific formulas proposed by Mennini et al. [7], however we felt that a more detailed analysis of indirect cost would require a larger population and was beyond the scope of the present study.

## Conclusions

This study confirms the high impact of migraine, especially in its chronic form, on the NHS and on patients themselves, both in terms of economic cost and of worsened quality of life.

Treatments or health interventions aimed at preventing conversion of EM into CM should be fostered and encouraged. More varied and specific treatments for CM, i.e. botulinum toxin or neuromodulation techniques, should be used and diffused to treat chronicity, to prevent relapses and, ultimately, to save significant resources on both the National Health System and patients themselves.

## Abbreviations

EM: Episodic migraine
CM: Chronic migraine
CH: Chronic headache
NHS: National Health System
DRG: Diagnosis Related Group
MOH: Medication overuse headache
TM: Transformed migraine
